# A drone-based survey for large, basking freshwater turtle species

**DOI:** 10.1371/journal.pone.0257720

**Published:** 2021-10-27

**Authors:** Amy P. Bogolin, Drew R. Davis, Richard J. Kline, Abdullah F. Rahman

**Affiliations:** 1 School of Earth, Environmental, and Marine Sciences, The University of Texas Rio Grande Valley, Brownsville, Texas, United States of America; 2 Department of Integrative Biology, Biodiversity Collections, The University of Texas at Austin, Austin, Texas, United States of America; Instituto Federal de Educacao Ciencia e Tecnologia Goiano - Campus Urutai, BRAZIL

## Abstract

Conservation concerns are increasing for numerous freshwater turtle species, including *Pseudemys gorzugi*, which has led to a call for more research. However, traditional sampling methodologies are often time consuming, labor intensive, and invasive, restricting the amount of data that can be collected. Biases of traditional sampling methods can further impair the quality of the data collected, and these shortfalls may discourage their use. The use of unmanned aerial vehicles (UAVs, drones) for conducting wildlife surveys has recently demonstrated the potential to bridge gaps in data collection by offering a less labor intensive, minimally invasive, and more efficient process. Photographs and video can be obtained by camera attachments during a drone flight and analyzed to determine population counts, abundance, and other types of data. In this study we developed a detailed protocol to survey for large, freshwater turtle species in an arid, riverine landscape. This protocol was implemented with a DJI Matrice 600 Pro drone and a SONY ILCE α6000 digital camera to determine *P*. *gorzugi* and sympatric turtle species occurrence across 42 sites in southwestern Texas, USA. The use of a large drone and high-resolution camera resulted in high identification percentages, demonstrating the potential of drones to survey for large, freshwater turtle species. Numerous advantages to drone-based surveys were identified as well as some challenges, which were addressed with additional refinement of the protocol. Our data highlight the utility of drones for conducting freshwater turtle surveys and provide a guideline to those considering implementing drone-mounted high-resolution cameras as a survey tool.

## Introduction

Turtles (order Testudines) have ancient origins, persisting for over 200 million years, but in recent decades have experienced widespread declines, with 61% of the 356 global turtle species considered threatened or extinct [1]. Loss of populations or species can detrimentally affect ecosystems [1], as several turtle species function as ecosystems engineers or keystone species [2–4], and many species play important roles in seed dispersal and germination, nutrient cycling, and bioturbation of soils [1]. Freshwater turtles can shape communities by altering environmental characteristics and increasing nutrient input [5], as well as altering prey species abundance [1]. Given the importance of turtles in the ecosystems where they are present, and their alarming rates of decline, it is important that efficient survey efforts are established to locate and monitor turtle populations.

Traditionally, freshwater turtles have been surveyed using trapping, visual surveys, or snorkeling surveys, but these methodologies are often time consuming, labor-intensive, and expensive, making it difficult to adequately assess turtle populations [6–9]. Furthermore, biases exist amongst some of these sampling methodologies. Differences due to bait type [10], sex [11], and trap design [10], can affect whether turtles enter traps. Additionally, the presence of turtles already in a trap can influence whether additional turtles enter or not [12]. Minimally invasive sampling methodologies such as visual surveys are often less effective than trapping, especially for elusive species, and limited to areas where water access is available [13–15]. Snorkel surveys require low turbidity and passable waterways and may not be suitable for fast-swimming species [16]. These shortfalls of traditional methodologies can lead to ineffective or inefficient survey efforts, draining limited resources and discouraging their use [17].

The use of small unmanned aerial vehicles (UAVs, drones) as an alternative survey method addresses some of the shortcomings of traditional turtle survey methods. With their increased availability and affordability, drone use by conservation workers and wildlife biologists has expanded. Drones have been used to light prescribed fires [18], to map water sources [19], to search for invasive plants [20], and to conduct wildlife surveys [21,22]. To date, numerous species have been successfully surveyed using drone-based methods, including large terrestrial and marine mammals [22–26], birds [27,28], and large aquatic reptiles [29]. Recently, freshwater turtle species have been added to the list of animals surveyed using drones [30–32]. Freshwater turtle drone surveys to date have been preliminary, with the potential use of drone surveys demonstrated by Biserkov and Lukanov [30] in a proof-of-concept study and expanded upon by Daniels [31], who compared drone surveys to visual surveys conducted with spotting scopes. Karcher [32] used drones to supplement the documentation of turtles basking on platforms but did not evaluate the use of drones as a sampling method. These previous studies provided the initial foundation for drone studies on freshwater turtles, but failed to report detailed flight parameters and conducted flights in variable, unrepeatable flight patterns, which limits the usefulness of these studies in informing future researchers who wish to conduct drone surveys. While still a relatively novel tool, the use of drone surveys for sea turtle detection has been well developed [29,39]. However, large body sizes, open habitat, and reduced habitat complexity increases the detectability of sea turtles and allow for flights to be conducted at greater heights [39]. In contrast, freshwater turtles have smaller body sizes and most live in structurally complex habitats which are visually restrictive due to tree cover and aquatic vegetation. These differences decrease the applicability of sea turtle drone survey parameters for freshwater turtle surveys.

Drones can be employed to conduct flights over a survey area, and cameras can be used to take photographs or videos, which are later analyzed for species abundance, threats, tracks, nesting sites, and other types of data [33–35]. Drones are relatively inexpensive when compared to traditional sampling methodologies, are less labor-intensive, and can often survey areas where access to sites is limited [26,33,36]. They also have the benefit of being less invasive, and documented wildlife responses to drone flights have been minimal [29,34,37] with flights as low as 7 m failing to disturb birds [38]. With continued technological advances and increased efficiency, drones are expected to become widely incorporated into wildlife surveys [34,36,39].

The aim of this study was to develop a drone-based survey to locate and detect Rio Grande Cooter (*Pseudemys gorzugi*) throughout a portion of its range in Texas, USA. *Pseudemys gorzugi* was the target species for this project due to recent conservation concerns [15,40,41], including an ongoing Species Status Assessment by the U.S. Fish and Wildlife Service [42]. However, two sympatric turtle species theRed-eared Slider (*Trachemys scripta elegans*) and Spiny Softshell (*Apalone spinifera*) were also documented and quantified during drone surveys, demonstrating the applicability of drones to survey for other freshwater turtle species as well. These three species were optimal targets for drone surveys given their large size and tendency to swim or bask near the surface, therefore aiding in their detection.

## Materials and methods

### Study sites

**Fig 1:**
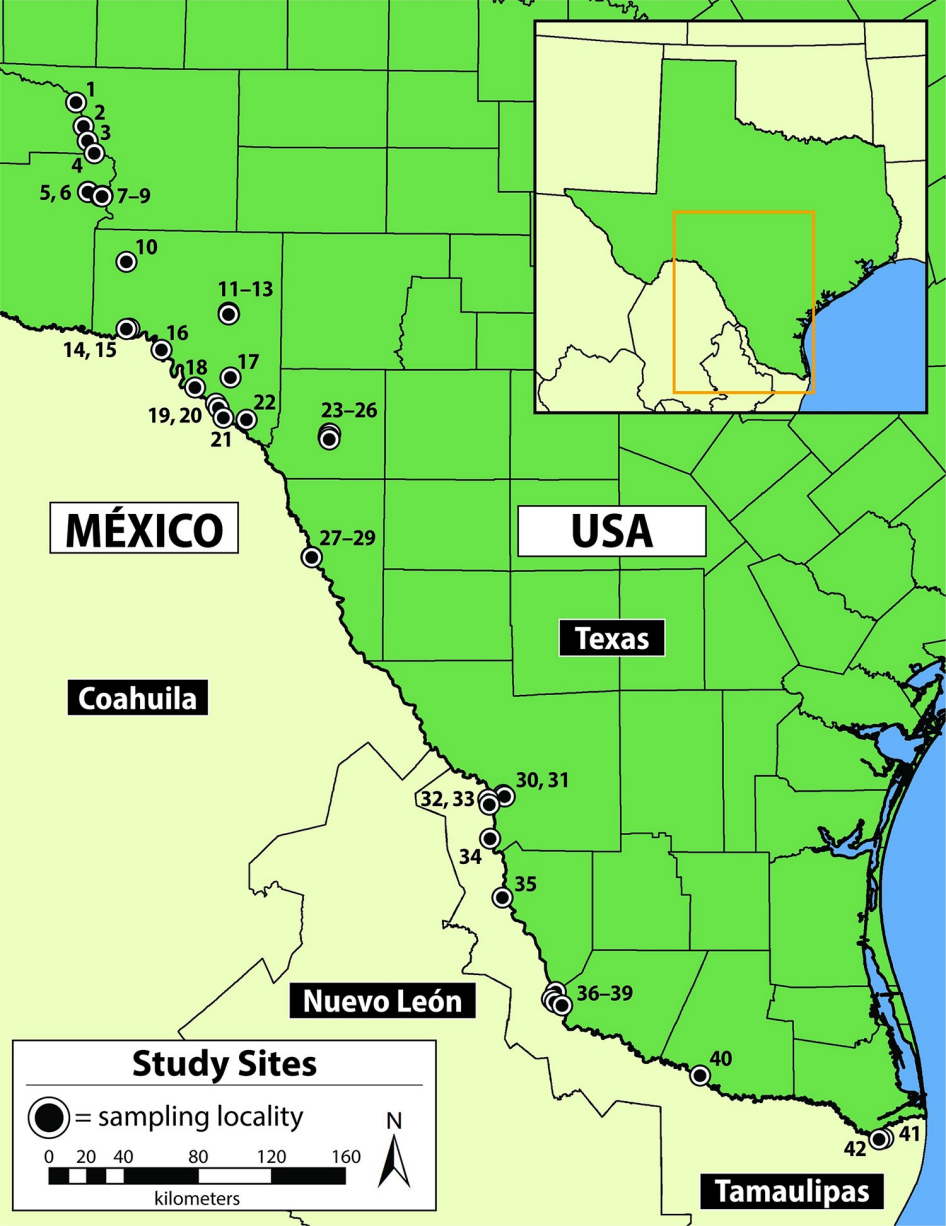
Map of drone sampling localities. Drone surveys for *Pseudemys gorzugi* (and sympatric turtles) occurred at 42 unique localities throughout southwestern Texas, USA. Site numbers correspond to those used in Tables 1 and S1.

Our study sites were located in southwestern Texas, USA, along the Rio Grande, lower Pecos, and Devils river watersheds. Drone surveys were conducted at 42 unique localities from the lower Pecos River in Pecos County to the lower Rio Grande in Cameron County (Fig 1, S1 Table). Locations surveyed encompass the majority of the recognized distribution of *Pseudemys gorzugi* in Texas (known populations near Big Bend National Park and the upper Pecos River near the New Mexico border were not included) and represent localities beyond this recognized distribution in hope of generating occurrence data at new localities. Twenty-six of the localities were sites where *P*. *gorzugi* is known to occur. The recognized distribution of *P*. *gorzugi* in this region is based off of vouchered museum specimens, literature reports, and photographs posted to citizen science platforms, and include occurrence records across a variety of habitats. A sizeable gap between our sampling sites existed along the Rio Grande between Eagle Pass and Laredo, Texas which was due to a lack of public lands and river access in this area. All sites were classified into habitat categories based upon habitat type (mainstem [n = 25], tributary [n = 12], or reservoir [n = 5] and water source (springs present [n = 12], or absent [n = 30] to better understand how drone results are influenced by habitat characteristics.

### Drone and camera setup

**Fig 2:**
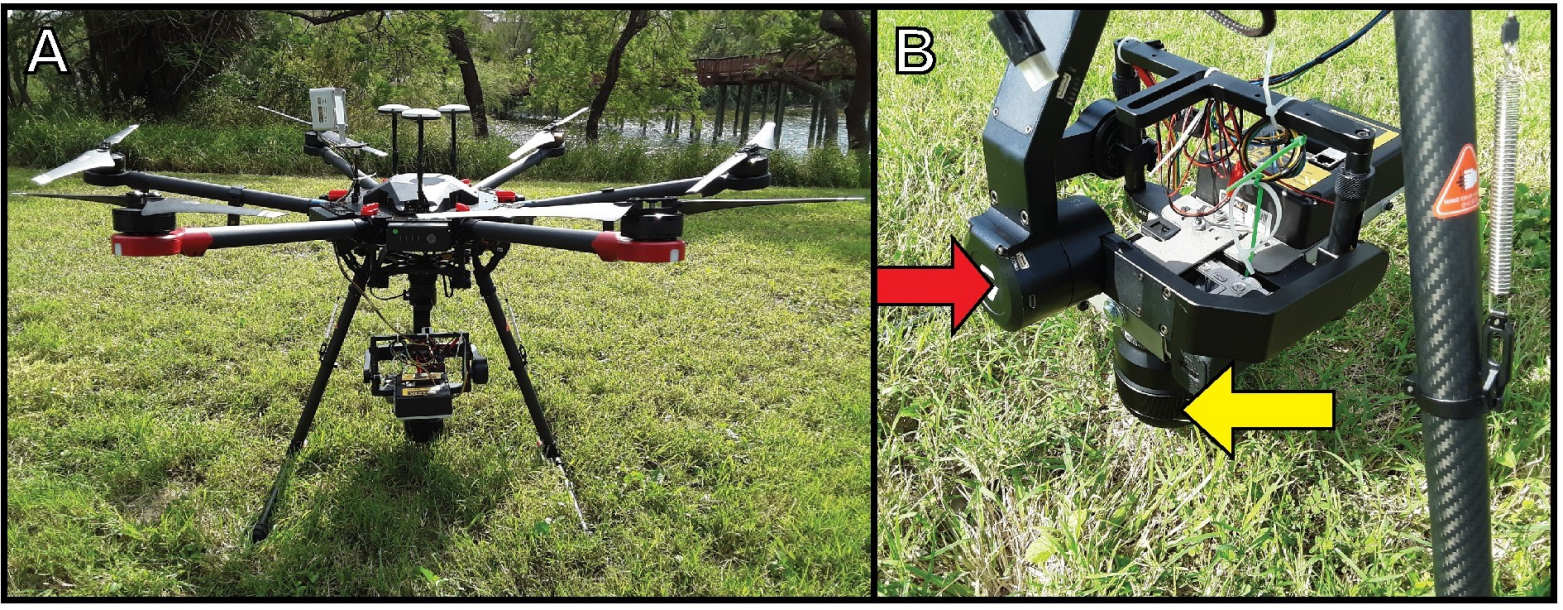
Drone and equipment used to conduct surveys for aquatic turtles. (A) DJI Matrice 600 Pro unmanned aerial vehicle with additional survey equipment attached; (B) Gremsy T-3 gimbal (red arrow) with the SONY digital camera (yellow arrow) attached.

A DJI Matrice 600 Pro unmanned aerial vehicle (cat. # CP.SB.000308, SZ DJI Technology Co., Ltd, Shenzhen, Guangdong, China) was used to conduct drone surveys (Fig 2A). The DJI Matrice 600 Pro is a LiPo 6S battery-powered, rotary-wing hexacopter, measuring 1668 × 1518 × 727 mm with propellers, frame arms, and GPS mount unfolded (including landing gear) and has a weight of 9.5 kg [43]. It has a battery life of 16 min when carrying a 6 kg payload, a maximum wind resistance of 8 m/s, and a maximum operating temperature of 40°C [43]. A Gremsy T-3 gimbal (cat. # Gremsy T3V3, Gremsy.com, Ho Chi Minh City, Vietnam) was attached and slightly modified to attach both a digital and multispectral camera (Fig 2B). This modification included installing two camera mounts and a weighted bar counterbalance to ensure that the payload was distributed evenly. A 24.3 MP SONY ILCE α6000 E-mount camera with APS-C sensor (cat. # ILCE-6000, SONY, Kōnan, Minato, Tokyo) with SONY FE 85 mm F1.8 prime lens (cat. # ILCE-6000, SONY, Kōnan, Minato, Tokyo) and a Platinum 67 mm UV lens filter (cat. # PT-MCUVF67, BBY Solutions, Inc, Richfield, Minnesota, USA) was used to locate, identify, and count turtles.

Two flight control programs (apps) were used to program and conduct the flights. The Maps Made Easy app (Drones Made Easy, San Diego, California, USA) was used for the majority of the flights. On occasion the DJI GSPro app (SZ DJI Technology Co., Ltd, Shenzhen, Guangdong, China) was utilized, as this app allowed for greater user input and a more accurate calculation of percentage overlap, which was beneficial for photo-stitching efforts. Drone surveys with this app consisted of two flights with a battery change, and battery limitations prevented it from being the primary app used. All flights were programmed prior to arrival at a study site, as internet access was required to download map imagery on the apps. Adjustments to the flight plan were made if needed in the field prior to the flight.

### Flight parameters tested

Flight parameters were developed through a series of initial test flights. Test flights occurred at 50 m above ground level (AGL) and at a maximum speed of 2.4 m/s, parameters that were based on recommendations for the multispectral camera to obtain a 10 ms exposure. However, turtles in images from the initial test flights were extremely difficult to detect due to the small size and lack of focus in the images taken at 50 m AGL. Further test flights occurred at 40 m AGL with these flights having no observable effects on turtle behavior. While some improvement was noted in the imagery, it was still not adequate to detect and identify turtles.

Optimal flight altitude and speed were arrived at by repeated trials, with 30 m AGL and a speed of 2.2 m/s determined as the best compromise for best image quality with minimal turtle disturbance. During test flights conducted at the Lozano Banco Resaca in Brownsville at 30 m AGL, the drone did not startle *Trachemys scripta elegans* from their aerial basking locations on multiples flights, and during test flights in the Rio Grande at Salineño 10–40% of observed turtles (*Pseudemys gorzugi* and *T*. *s*. *elegans*) left their basking substrate when the drone flew overhead only on a few occasions (30% of flights); however in most instances the drone did not appear to disturb basking turtles. Additionally, while observing responses to the drone, turtles that left basking locations remained near these locations and were able to be identified and counted through drone imagery. Flights below 30 m AGL were also conducted at the Lozano Banco Resaca on a few instances, again with no effect on basking *T*. *s*. *elegans*. Though these lower flights may have been feasible to conduct in the field, reductions to the total flight area would have occurred.

SONY camera settings were also arrived at through repeated trials. In total, 216 different camera settings were tested with F-stop ranging from 4.5–16, ISO from 100–400, and a shutter speed from 1/640–1/1250 s. Additionally, the manual focus had to be set using the manual focus distance prompt in the lens viewer. Tested focus distances ranged from 26–30 m. The optimal and final settings that were chosen were a manual focus camera prompt distance of 29 m, ISO at 320, F-stop at 6.3, and the shutter speed at 1/1000.

Initially, camera triggering was controlled through the PlayMemories Time-lapse app v. 3.40 (SONY, Kōnan, Minato, Tokyo) on a 2-s interval. This was reduced to 1 s to ensure that the entire survey area was being covered. Later, a GeoSnap Express (cat. # GSS-EXP-SLR-STD, Field of View LLC, Fargo, ND, USA) was attached to the digital camera to provide GPS locations for photographs to use in post-flight processing and analysis, and camera triggering was switched to this platform. With the GeoSnap Express, the camera was triggered to take a photograph every 2 m to reduce the number of photographs taken while the drone was launching and landing. GPS error prevented consistent triggering of the camera, so settings were then switched back to a 1-s interval and triggered through the GeoSnap Express. Photographs were taken in both JPEG and RAW format and stored on SD cards for later analysis.

**Fig 3:**
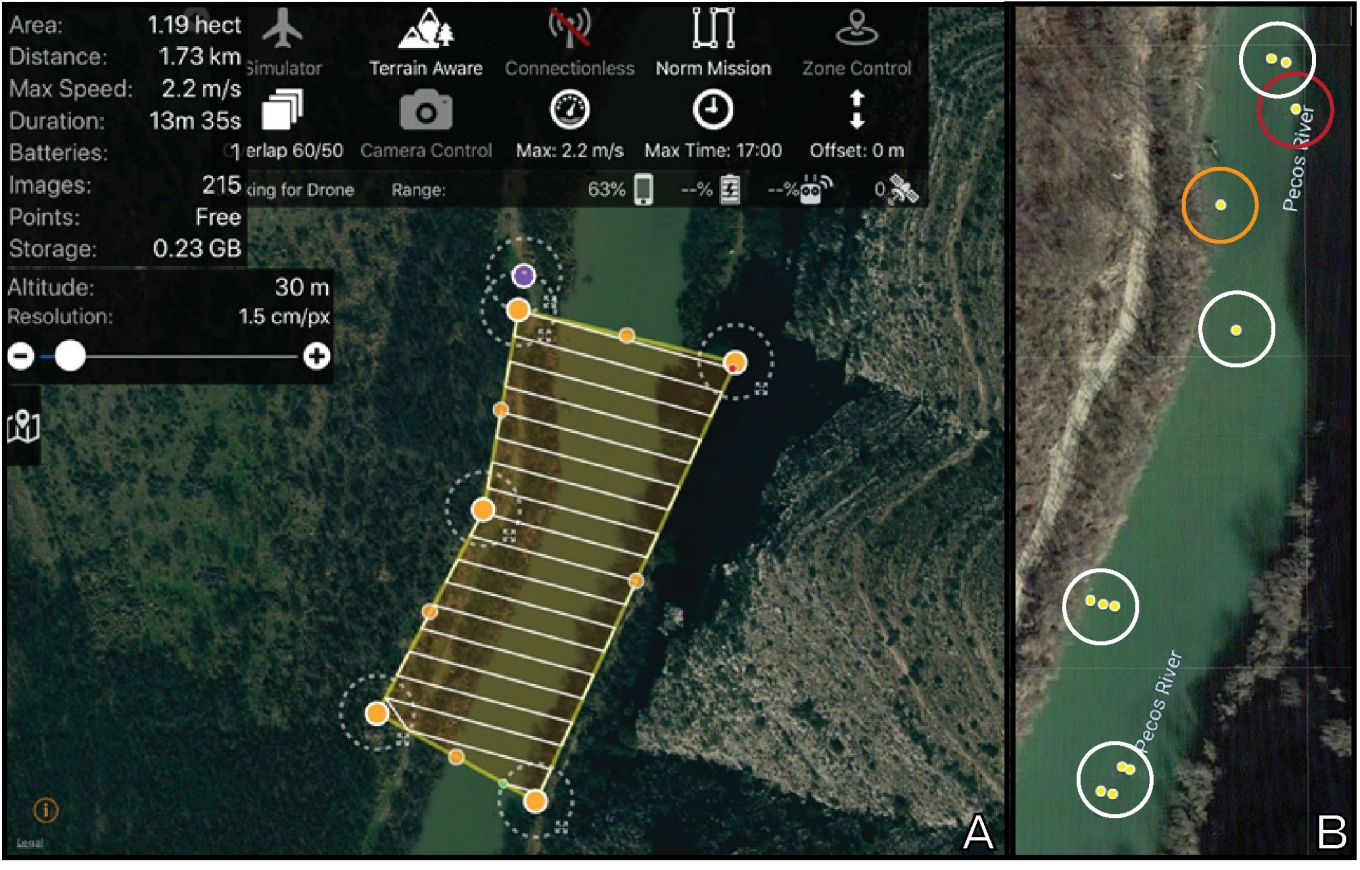
Programs used to conduct drone flights and process drone imagery. (A) Screenshot from the Maps Made Easy app that was used to conduct the majority of the drone flights during this project. The projected flight path and flight parameters are depicted for a flight on 10 August 2019 along the Pecos River, 0.8 river km upstream of confluence with Independence Creek, Crockett County. (B) Map showing locations of turtle detections during a drone survey for this same flight. By examining GPS locations of photographs containing turtles, the relative location of the detections could be determined to assist in turtle quantification. Turtles were captured in 12 photographs taken during this drone survey (yellow dots). However, multiple images often captured the same individual turtle, indicated by a circle (orange = *Pseudemys gorzugi*; red = *Trachemys scripta elegans*; white = *Apalone spinifera*).

Flights were conducted in linear transects and were perpendicular to the direction of flow in lotic systems to assist in photo-stitching. Drone flights were conducted from 9 March–23 October 2019 between 0800 and 1830 h. The entire study area was surveyed, totaling to ca. 1.2 ha with a 10 m border around the waterbody (Fig 3A). This was the maximum area that could be surveyed with one set of batteries. Survey transect frontal overlap for photos ranged from 50–60% with maximum speed ranging from 2.2–2.5 m/s. Flights conducted with the DJI GSPro app had a side overlap of 50%. Due to battery limitations, drone surveys with this app consisted of two flights. No specialized launching equipment was required and in most instances level ground was located at the survey site and used as a launch point. Frequently, those launch points were parking areas. On two occasions it was not possible to launch the drone from ground-level, resulting in one flight at 43 m and another at 73 m. In these instances, the drone was launched from cliffs above the survey area. Air temperature was recorded at the time of drone survey using AccuWeather (https://www.accuweather.com/) as were weather conditions. All drone flights were conducted by APB and under a Federal Aviation Administration remote pilot license (certificate # 4189203).

### Photo analyses

After completing the flight, photographs from the SONY camera were individually and manually analyzed to detect and identify turtles. To differentiate between species, pattern and morphology of the head and carapace were used to identify turtle species. *Pseudemys gorzugi* was identified by distinctive yellow bands on top of the head, red-orange webbing between the toes, and concentric circles on their carapace. *Trachemys scripta elegans* was identified by red bands on the head by the tympana and yellow bands that often extend down the sides of their carapace. *Apalone spinifera* was identified by their light gray or tan color, narrow head, elongated snout, and the vertebrae of their backbone that are visible through their leathery carapace. In cases when an individual turtle was unable to be identified to species it was classified as unknown.

Two methods were attempted to detect and identify turtles in photographs. Agisoft Metashape photogrammetry software (Agisoft LLC, St. Petersburg, Russia) was used to create a photomosaic for each test site and additionally, photographs containing turtles were uploaded into Google Maps (Google LLC, Mountain View, California, USA) utilizing their GPS stamps to determine their locations relative to other photographs containing turtles (Fig 3B). Multiple photographs of the same turtle occurred due to the high overlap between transects and high photograph interval rate. Adjacent photographs were visually examined to determine if any of the turtles present were duplicates from other photographs by looking at individual characteristics of the turtles such as size, sex, unique markings, as well as their activity and location relative to their surroundings. Most turtles exhibited minimal movement during the survey, allowing for easy recognition of individuals through the use of landmarks. Challenges mostly arose in locations where large numbers of turtles were swimming in open water, leading to some challenges in individual identification during the two flights with the highest detection numbers. After accounting for duplicate turtles, final counts were determined for each species. Photogrammetry software often failed to stitch imagery of open water, which constituted a large portion of our study sites, and on several occasions produced photo-mosaics that were fragments of the site by omitting large portions of the flight area. Additionally, in instances where photographs were successfully stitched, many turtles that were visible in original photos were no longer apparent, likely due to smoothing processes that occurred during imagery mosaicking [44]. Due to these challenges, photo-stitching was not used to quantify and identify turtles, and the GS Pro app was no longer advantageous, resulting in the use of the Maps Made Easy app for flights.

### Quantifying the results of drone surveys and efficacy of its use

Total turtle detections from drone-based imagery was recorded by species. Identification percentages were averaged for each site and for the entire study for comparison. Observations of turtle behavior and other species present in drone imagery were noted. Means for all analyses are reported as mean (± 1 SD). The data for this project did not meet the assumptions of parametric analyses, and as a result Kruskal-Wallis and Mann-Whitney U-tests were used for analysis. All analyses were conducted in JMP v14 statistical software (SAS Institute, Cary, NC, USA). All raw data collected as part of this study is publicly accessible through Open Science Framework (https://bit.ly/3ckl17ar).

### Ethics statement

All research was conducted under a Texas Parks and Wildlife (TPWD) Scientific Permit for Research (SPR-1018-294), TPWD State Park Scientific Study Permit (2019_R2_RGV_02), TPWD Aerial Wildlife and Exotic Animal Management Permit (M-1603), NPS Scientific Research and Collecting Permit (AMIS-2018-SCI-0007), The Nature Conservancy (Texas Chapter) Scientific Investigation and Collection Permit, International Boundary and Water Commission (IBWC) U.S. Section Permit (USIBWC-19-2-0011), Certificate of Waiver or Authorization (2019-P107-CSA-10089), and a University of Texas Rio Grande Valley Institutional Animal Care and Use Committee protocol (AUP# 18–28). Drone flights were conducted at 30 m AGL to minimize disturbance to wildlife.

## Results

Seventy-three drone surveys were conducted at 42 unique localities throughout the sampling period. While an effort was undertaken to obtain an equal number of visits among localities, logistical challenges and unfavorable weather occasionally prevented drone flights, resulting in 1–3 surveys being conducted at each site (Table 1). A total of 84,441 photographs were collected from drone surveys with 1,444 photographs containing at least one turtle. This resulted in 640 turtle detections, including *Pseudemys gorzugi* (n = 307), *Trachemys scripta elegans* (n = 93), *Apalone spinifera* (n = 89), and unidentifiable turtles (n = 151). The average species identification percentage of turtles depicted in drone-based imagery throughout this study was 82.3% (± 27.8).

**Table 1:** Turtle detections and identification percentages from drone surveys.

Site #	# of Visits	*Pseudemys gorzugi*	*Trachemys scripta elegans*	*Apalone spinifera*	Unknown	ID %
1	2	0 (± 0)	1.0 (± 0)	0.5 (± 0.7)	0 (± 0)	100.0 (± 0)
2	2	0 (± 0)	0.5 (± 0.7)	0 (± 0)	0 (± 0)	100.0 (± N/A)
3	1	0 (± N/A)	2.0 (± N/A)	0 (± N/A)	0 (± N/A)	100.0 (± N/A)
4	1	0 (± N/A)	1.0 (± N/A)	0 (± N/A)	0 (± N/A)	100.0 (± N/A)
5	2	4.5 (± 3.5)	1.0 (± 1.4)	2.0 (± 1.4)	0 (± 0)	100.0 (± 0)
6	1	3.0 (± N/A)	0 (± N/A)	0 (± N/A)	0 (± N/A)	100.0 (± N/A)
7	1	1.0 (± N/A)	1.0 (± N/A)	4.0 (± N/A)	0 (± N/A)	100.0 (± N/A)
8	2	0 (± 0)	0 (± 0)	0.5 (± 0.7)	0 (± 0)	100.0 (± N/A)
9	2	1.0 (± 0)	0 (± 0)	2.5 (± 0.7)	0 (± 0)	100.0 (± 0)
10	2	3.0 (± 1.4)	0 (± 0)	0 (± 0)	0 (± 0)	100.0 (± 0)
11	2	1.5 (± 2.1)	0.5 (± 0.7)	0 (± 0)	0.5 (± 0.7)	80.0 (± N/A)
12	2	0 (± 0)	0 (± 0)	0 (± 0)	0.5 (± 0.7)	0 (± N/A)
13	3	29.0 (± 22.5)	0 (± 0)	0 (± 0)	5.7 (± 4.6)	83.9 (± 1.0)
14	1	0 (± N/A)	0 (± N/A)	0 (± N/A)	0 (± N/A)	-
15	1	4.0 (± N/A)	0 (± N/A)	2.0 (± N/A)	13.0 (± N/A)	31.6 (± N/A)
16	1	1.0 (± N/A)	0 (± N/A)	5.0 (± N/A)	0 (± N/A)	100.0 (± N/A)
17	2	0 (± 0)	0 (± 0)	0 (± 0)	1 (± 1.4)	0 (± N/A)
18	2	0 (± 0)	0 (± 0)	0 (± 0)	0 (± 0)	-
19	1	56.0 (± N/A)	0 (± N/A)	8.0 (± N/A)	16.0 (± N/A)	80.0 (± N/A)
20	1	0 (± N/A)	3.0 (± N/A)	0 (± N/A)	0 (± N/A)	100.0 (± N/A)
21	2	0 (± 0)	0 (± 0)	0 (± 0)	0 (± 0)	-
22	3	11.7 (± 9.3)	5.3 (± 3.2)	10.7 (± 4.6)	9.3 (± 1.5)	71.3 (± 13.2)
23	3	9.0 (± 1.7)	3.0 (± 3.0)	0 (± 0)	12 (± 8.0)	53.4 (± 24.6)
24	1	0 (± N/A)	0 (± N/A)	0 (± N/A)	0 (± N/A)	-
25	3	5.0 (± 3.6)	0 (± 0)	0.3 (± 0.6)	2.3 (± 2.1)	78.0 (± 22.2)
26	2	6.0 (± 4.2)	3.5 (± 0.7)	1.0 (± 0)	2.0 (± 0)	82.6 (± 6.9)
27	2	19.0 (± 14.1)	2.5 (± 2.1)	4.0 (± 1.4)	4.5 (± 3.5)	86.3 (± 5.3)
28	1	0 (± N/A)	0 (± N/A)	2.0 (± N/A)	0 (± N/A)	100.0 (± N/A)
29	1	1.0 (± N/A)	19.0 (± N/A)	7.0 (± N/A)	10 (± N/A)	73.0 (± N/A)
30	1	0 (± N/A)	1.0 (± N/A)	1.0 (± N/A)	0 (± N/A)	100.0 (± N/A)
31	1	0 (± N/A)	1.0 (± N/A)	1.0 (± N/A)	0 (± N/A)	100.0 (± N/A)
32	3	2.0 (± 1.7)	0 (± 0)	0 (± 0)	0.7 (± 1.2)	80.0 (± 28.3)
33	2	0 (± 0)	0 (± 0)	1.0 (± 1.4)	0 (± 0)	100.0 (± N/A)
34	2	0 (± 0)	0 (± 0)	0 (± 0)	0 (± 0)	-
35	1	0 (± N/A)	0 (± N/A)	0 (± N/A)	0 (± N/A)	-
36	2	0 (± 0)	2.0 (± 2.8)	0.5 (± 0.7)	1.0 (± 0)	41.7 (± 58.9)
37	3	0 (± 0)	1.3 (± 2.3)	0 (± 0)	1.0 (± 1.7)	57.1 (± N/A)
38	1	0 (± N/A)	0 (± N/A)	0 (± N/A)	0 (± N/A)	-
39	2	0.5 (± 0.7)	4.0 (± 2.8)	1.0 (± 1.4)	0 (± 0)	100.0 (± 0)
40	2	0 (± 0)	0 (± 0)	0 (± 0)	0 (± 0)	-
41	1	0 (± N/A)	2.0 (± N/A)	0 (± N/A)	0 (± N/A)	100.0 (± N/A)
42	2	0 (± 0)	0.5 (± 0.7)	0 (± 0)	0 (± 0)	100.0 (± N/A)
**Total**	**73**	**3.8 (± 10.0)**	**1.3 (± 3.1)**	**1.3 (± 2.4)**	**1.9 (± 4.0)**	**82.3 (± 27.8)**

Average turtle detections per site (± 1 SD) as a result of drone surveys conducted at sampling sites. Results are broken down by species identified (*Pseudemys gorzugi*, *Trachemys scripta elegans*, and *Apalone spinifera*) with unidentifiable turtles classified as unknown. Average identification percentages per site and number of site visits are also displayed. Individual identification percentages from multiple visits to a single site were only averaged if at least one turtle was detected during that visit. Site numbers corresponds to sites shown in Fig 1 and S1 Table.

Substantial habitat variation occurred throughout the study sites, with flights occurring over lentic and lotic systems, in areas with various degrees of shoreline vegetation and canopy cover, and in pristine and highly disturbed environments (S1 Table). Drone imagery was able to successfully document turtles at these locations despite habitat differences, with the only challenge appearing where we were unable to access ground level to launch the drone. Two flights, one at Pump Canyon, Langtry (Site 16), and the other at TNC Dolan Falls Preserve, Devils River, Dolan Falls (Site 13), occurred at altitudes above 30 m AGL due to challenges accessing ground level. At Pump Canyon, the flight occurred at 70 m AGL which caused image resolution to be low and identifications percentages to be low (Table 1). The flight at Dolan Falls occurred at 43 m AGL, and while higher than other flights, this increased altitude did not substantially decrease image resolution, resulting in a similar identification percentage to flights at 30 m AGL (Table 1). No significant difference was observed in the number of turtle detections among mainstem, tributary, or reservoir habitats (Kruskal-Wallis test: H = 4.13, df = 2, p = 0.127), and we detected significantly more turtles at spring sites than those where springs were absent (Mann Whitney U-test: H = 3.95, df = 1, p = 0.047).

**Fig 4:**
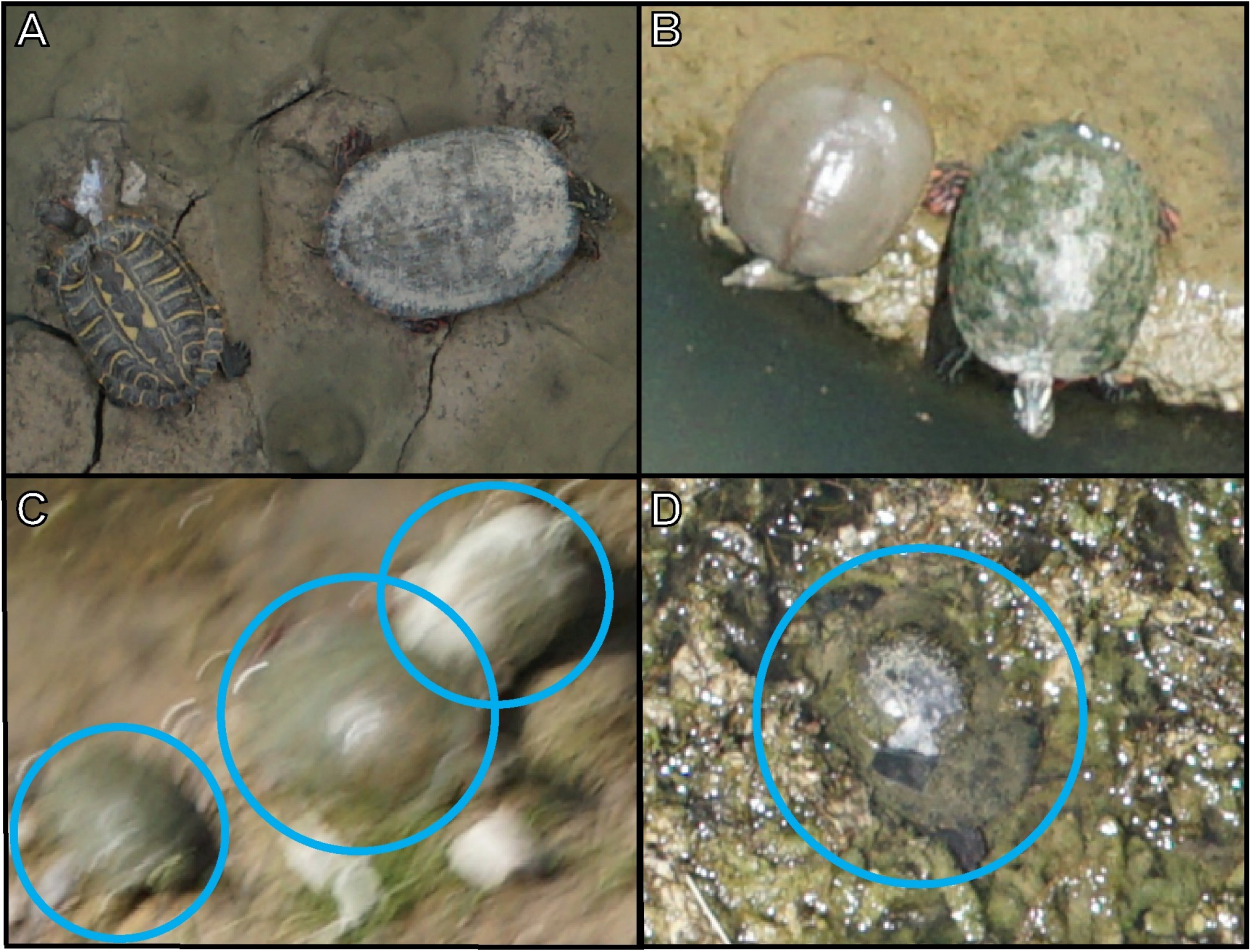
Magnified drone imagery of turtles. Magnified drone images depicting the species of turtles identified throughout this study and instances where turtles were unable to be identified. (A) *Trachemys scripta elegans* on left and *Pseudemys gorzugi* on right basking in the Rio Grande, near Salineño, Starr County; (B) *Apalone spinifera* on left and *P*. *gorzugi* on right basking at the Eagle Pass Golf Course, spillway into Rio Grande, Maverick County; (C) out of focus, unidentified turtles (circles) basking at the Eagle Pass Golf Course, spillway into Rio Grande, Maverick County; (D) obscured, unidentified turtle (circle) at TNC Dolan Falls Preserve, Devils River, upstream of confluence with Dolan Creek.

Each photograph at 30 m AGL covered an area of 46 m^2^ (8.29 × 5.53 m) with a pixel size of 1.4 × 1.4 mm, providing photograph resolution that was sufficient to detect species-specific characteristics of aquatic turtles (Fig 4A and 4B). Instances where a species identification could not be assigned were often because photos were out of focus due to wind moving the camera (Fig 4C) and turtles being obscured by turbid water, vegetation, or shadows (Fig 4D). Additionally, the red markings on *T*. *s*. *elegans* are often faded in melanistic males, which likely led to the categorization of some of these turtles as unidentifiable.

Successful survey flights occurred in variable weather conditions from 18–39°C (mean ± SD = 30 ± 5°C), on clear and overcast days, and in wind speeds up to 8 m/s. Weather conditions experienced during drone surveys did not appear to influence detections, with detections both occurring on clear and overcast days, and no observed relationship between ari temperature and number of detections (F_1,71_ = 0.36; r^2^ = 0.005; p = 0.5). All flights were conducted between 0900–1700 h to avoid periods of low light, and turtle detections occurred throughout this entire time period. Drone flights conducted with the Maps Made Easy app were on average 14 min 25 s (± 1 min 15 s) in duration with a range of 9 min 23 s to 16 min 25 s and both apps covered on average a survey area of 1.19 (± 0.21) ha. Average detections for each species over the 42 sites were 3.76 (± 9.98) *P*. *gorzugi*, 1.31 (± 3.08) *T*. *s*. *elegans*, 1.29 (± 2.42) *A*. *spinifera*, and 1.89 (± 4.05) unknown turtles (Table 1). The highest number of turtle detections that resulted from a single drone survey was 80 turtles, with 64 turtles identified to species (80% identification; Site 19; Table 1).

**Fig 5:**
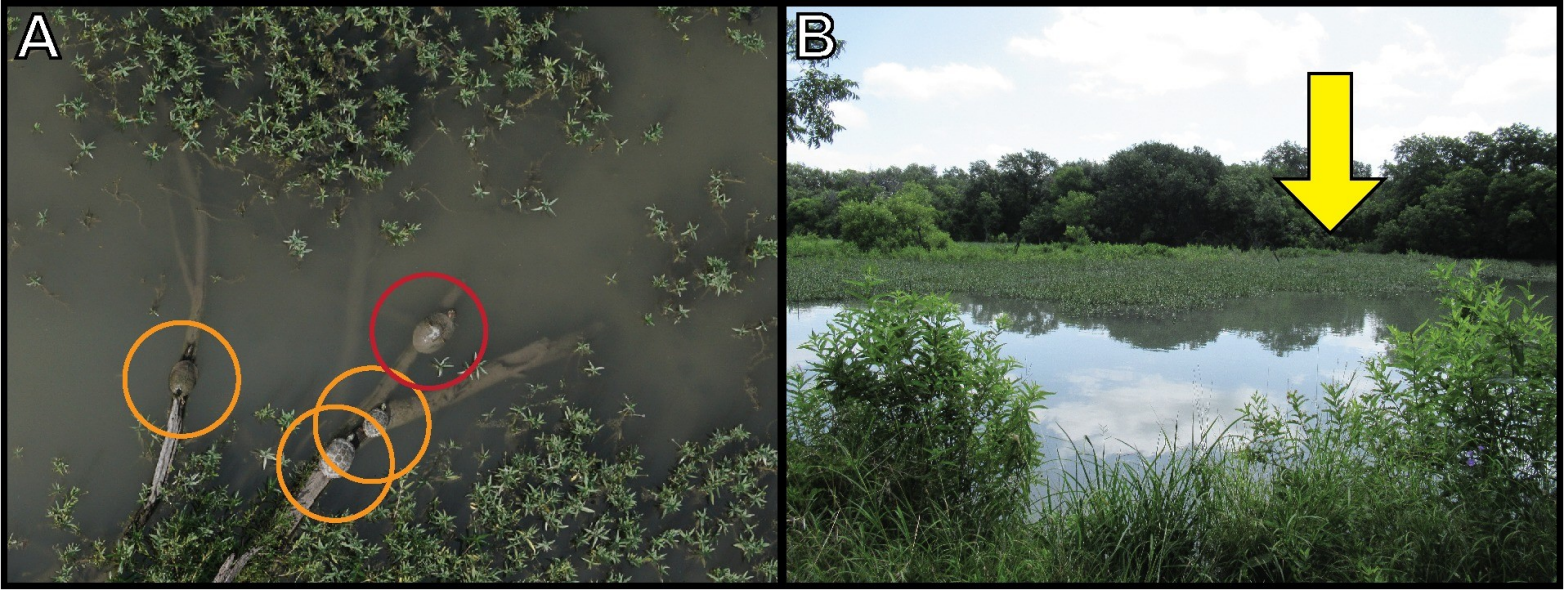
Comparison of aerial and shoreline viewpoint. (A) Drone imagery was able to detect three *Pseudemys gorzugi* (orange circles) and one *Trachemys scripta elegans* (red circle) basking on a log at Fort Clark Springs, Las Moras Creek, Buzzard Roost, Kinney County that were not visible from shore; (B) View of the site from shoreline with the log and turtles (yellow arrow) obscured by emergent vegetation.

With its unique aerial viewpoint, the drone was consistently able to document turtles that were not visible from shore (Fig 5), including the first detection of *P*. *gorzugi* in a previously unreported county [45]. Numerous identifiable behaviors of *P*. *gorzugi* were also documented, including mass basking of 26 *P*. *gorzugi* sharing a single basking rock, subaerial basking, courtship, and foraging with drone imagery showing an adult male *P*. *gorzugi* approach and begin to consume a piece of aquatic vegetation floating on the surface of the water through a series of photographs (S1 Fig). Throughout the study, numerous species of non-target wildlife were documented in drone imagery, including several species of birds, fish, and invertebrates (S2 Fig).

## Discussion

Drone surveys resulted in high-quality imagery with minimal disturbance to turtles and other wildlife. The superiority of the drone’s aerial vantage to that of a shoreline viewpoint for collecting population count data that we observed was also noted in Hodgson et al. [46]. A high number of overall detections demonstrates the ability of drone-based surveys to locate turtles in their natural environment. While previous freshwater turtle drone surveys have faced challenges with turtle identification, particularly for turtles swimming in water [31], using a higher resolution camera led to high identification percentages for this study. These high identification percentages further demonstrated the ability of drone-based surveys to produce quality data, which is essential for species-specific surveys and management. These characteristics are crucial for wildlife surveys [47], and drone-based surveys resulted in high overall detections and high identification percentages, demonstrating its applicability.

Observations of turtle behavior through drone imagery provided supplemental information on turtle activity. Basking of freshwater turtles has been previously documented through drone imagery by Biserkov and Lukanov [30], Daniels [31], and Karcher [32]; however, we are not aware of previous drone surveys detecting such high numbers of turtles basking together as we had: on 9 March 2019 at the Eagle Pass Golf Course, spillway into Rio Grande (Site 27), we observed 26 adult *P*. *gorzugi* basking on a single rock. This site, a lentic spillway along the mainstem of the Rio Grande was relatively shallow with numerous basking locations. These basking turtles would likely have been startled when approaching to conduct a visual survey and may have been challenging to count from a shoreline perspective. Subaerial basking, which is a thermoregulatory behavior of freshwater turtles in warm environments where individuals bask on top of algal mats and other aquatic vegetation [48] was frequently observed, occurring on 12 of the 23 drone surveys at 14 sites with algal or aquatic vegetation mats suggesting that this is a highly used thermoregulatory behavior across the region. Observations of courting behaviors provided data on the timing of reproductive ecology for *A*. *spinifera* and *P*. *gorzugi* as similarly seen in drone surveys of Green Sea Turtles (*Chelonia mydas*) conducted by Bevan et al. [49]. Drone imagery has successfully documented foraging in other species, including sea turtles [29], Pygmy Blue Whales [50], and American Black Bears [51], showing the potential of drone surveys to generate additional information on species’ diet, which was reflected in this study. Documentation of various behaviors is not unique to drone surveys, as visual and snorkeling surveys can also document behaviors [52–54]. However, trapping surveys, while commonly used, fail to result in behavioral observations. Additionally, the aerial viewpoint may provide superior observations than visual and snorkel surveys due to reduced sun glare, less obstruction from shoreline vegetation, and a decreased likelihood of the observer’s presence altering turtle behavior, further demonstrating the benefit of drones in wildlife surveys.

The documentation of non-target wildlife confirms the application to survey other species of wildlife through aerial surveys, which has been noted in other studies [26,55]. Kudo et al. [38] found that seabirds, a non-target species, seemed undisturbed by the presence of a small remote-control helicopter flying overhead while surveying for salmon. On one occasion during the present study, an Osprey (*Pandion haliaetus*) caught and consumed a fish while the drone was conducting a survey directly above it, supporting our perception that small unmanned aerial vehicles have a minimal effect on animal behavior.

While drone-based surveys have many benefits, several challenges have yet to be fully addressed. Among these hurdles, some are technical, and others are of bureaucratic nature. Numerous approvals and permissions are required for drone surveys which can be a time-consuming process. The U.S. Federal Aviation Administration requires drone operators to obtain a Remote Pilot License, which requires passing an aeronautical knowledge exam [56]. Federal agencies require an additional lengthy permitting process for drone aspects of studies, and the U.S. Fish and Wildlife Service currently has a no-drone policy which denied us access to survey on National Wildlife Refuges [57]. Photographing wildlife through aerial methods required permits as well [58] and obtaining all these permits took us several months. Additionally, many of our study sites occurred on the international border with Mexico, and additional permits would have been needed to survey the entire width of the Rio Grande (the midline of the river is the international boundary).

The temperature threshold for the drone and digital cameras was 40°C [59,60], however, the equipment often experienced temperatures above this threshold, with post-flight battery temperatures in the low 40°C range on several occasions. Batteries would retain heat for an extended time, requiring cooling in air conditioning for several hours before they were able to be recharged. On several occasions the tablet used to conduct drone flights overheated and shut off, requiring the drone to be piloted with the tablet inside an air-conditioned vehicle. There are several actions that can be taken to mitigate environmental challenges. Limiting drone flights to cooler periods of the day and year when possible or switching equipment to another brand with a higher tolerance for heat [61] can be beneficial when operating in high temperature conditions. We found keeping equipment in the cab of an air-conditioned vehicle during transport to be beneficial in prolonging its use in high temperatures. Additionally, Duffy et al. [62] offered suggestions to mitigate the difficulties of drone operation in a variety of challenging environments and provides advice on some weather-related limitations.

We found the drone to be equally effective at detecting turtles in mainstem, tributary, and reservoir systems with no significant difference among these categories. However, we did observe a difference between spring-fed and non-spring-fed sites. It is unknown whether more turtles were detected at spring sites due to lower turbidity that was generally characteristic of these sites, or ir this is the result of a habitat preference exhibited by these turtle species. Certain habitat conditions such as turbid water and canopy cover can obscure turtles and prevent their detection in drone-based surveys [63]. While most of our study sites were located in areas with minimal canopy cover, at our more wooded sites, there were instances where the water was obscured by vegetation and likely resulted in missed detections. Missed detections also likely occurred in areas with turbid waters where turtles were lower in the water column. For drone surveys to provide accurate abundance data, these habitat conditions should be further investigated using controlled experiments with models and methodology comparison studies to determine the relationship between detections and population size in unfavorable conditions. Furthermore, our study focused on large, freshwater turtles that are typically observed basking or swimming near the water surface [48,64,65]. These species’ large size and behavior facilitated detections through drone surveys, minimizing availability bias. For other aquatic turtles such as *Sternotherus* and *Kinosternon* species, small body sizes and bottom-dwelling habits may prevent their detection through drone surveys [66]. Additionally, some larger aquatic species, such as *Chelydra* and *Macrochelys*, may be challenging to detect due to infrequent basking and their tendency to stay among the substrate at the bottom of the water column [67,68]. While species limitations exist, this study demonstrates the utility of drone-based surveys of large freshwater turtles, particularly basking species.

With time and experience, we were able to address most drone-related issues, and technological advancements should solve remaining issues as drones continue to be implemented in scientific studies. Limitations such as short battery life are being continually addressed, with newer models offering longer flight times than previous models [69], which permits larger survey areas and increases the practicality of these surveys. Additionally, recent advancements have increased wind resistance, provide rain resistance, allow for a larger range of operating temperatures, and provide obstacle avoidance [69], increasing the applicability of drones under a wider range of environmental conditions. We acknowledge the potential of drone surveys to document large, freshwater turtle species and believe that implementation should be feasible as technology continually progresses. Discovering optimal camera and flight parameters is a time-consuming and labor-intensive process as Joyce et al. [70] previously noted, particularly in marine and freshwater environments which involve the complexities of working over water. Multiple sets of camera settings could be developed for different conditions, such as when full sun is directly overhead or in cloudy conditions, which could increase focus and improve identification percentage; however, we determined the most optimal camera settings that could be used in a variety of light conditions determined by weather and time of day. The drone protocol depicted in this study (S1 Protocol) can be tailored to different environments, and we encourage further exploration into its different applications as a management tool for wildlife conservation.

## Supporting information

S1 Fig Magnified drone imagery of turtle behaviors. (A) 26 *Pseudemys gorzugi* basking on one rock at Eagle Pass Golf Course, spillway into Rio Grande, Maverick County. An additional *P*. *gorzugi* is seen swimming towards the rock for 27 *P*. *gorzugi* total in this image; (B) Subaerial basking of *P*. *gorzugi* and *Trachemys scripta elegans* on aquatic vegetation at Del Rio, San Felipe Springs Golf Course, San Felipe Creek, Val Verde County; (C) Two *Apalone spinifera* exhibiting courting behaviors in the Rio Grande, spillway below Amistad Dam, Val Verde County; (D) *Pseudemys gorzugi* seen foraging on aquatic vegetation in TNC Dolan Falls Preserve, Devils River, Dolan Falls, Val Verde County.
(TIF)

S2 Fig Magnified drone imagery of non-target species. Examples of non-target species that were photographed during surveys, all of which seemed unaffected by the presence of the drone: (A) Five Black-bellied Whistling Ducks (*Dendrocygna autumnalis*) perched on a log at Fort Clark Springs, Las Moras Creek, Buzzard Roost, Kinney County; (B) Native and introduced fish (Cypriniformes) swimming at Fort Clark Springs, Headwater Pond, Kinney County; (C) Monarch Butterfly (*Danaus plexippus*) flying over the Pecos River, 0.3 km upstream of confluence with Independence Creek, Crockett County; and (D) Dragonfly (Odonata) flying above the Pecos River, at Pandale Crossing.
(TIF)

S1 Table Sampling locality information and habitat characteristics. Site information and habitat characterization data for the 42 localities where drone surveys were conducted for *Pseudemys gorzugi* throughout southwestern Texas, USA. Sites were classified as waterbody type (M = mainstream, T = tributary, R = reservoir), spring-fed (Y or N), presence of aquatic vegetation mats (Y or N), woody debris (Y or N), trees (Y or N) and shoreline vegetation of ca. 2 m or greater (Y or N). For all categories Y = yes and N = no. Site numbers correspond to Table 1 and Fig 1.
(DOCX)

S1 Protocol Drone survey protocol. Protocol detailing drone flight preparations, instructions for flight, field checking data, and image analysis that was used for this study. This can be used as a guideline and modified to be appropriate for different equipment, environments, and sampling conditions.
(DOCX)
