# Supplementary figures and images for: A drone-based survey for large, basking freshwater turtle species
Source: PLoS One. 2021 Oct 27;16(10):e0257720. doi: 10.1371/journal.pone.0257720 (PMC8550609; doi:10.1371/journal.pone.0257720)

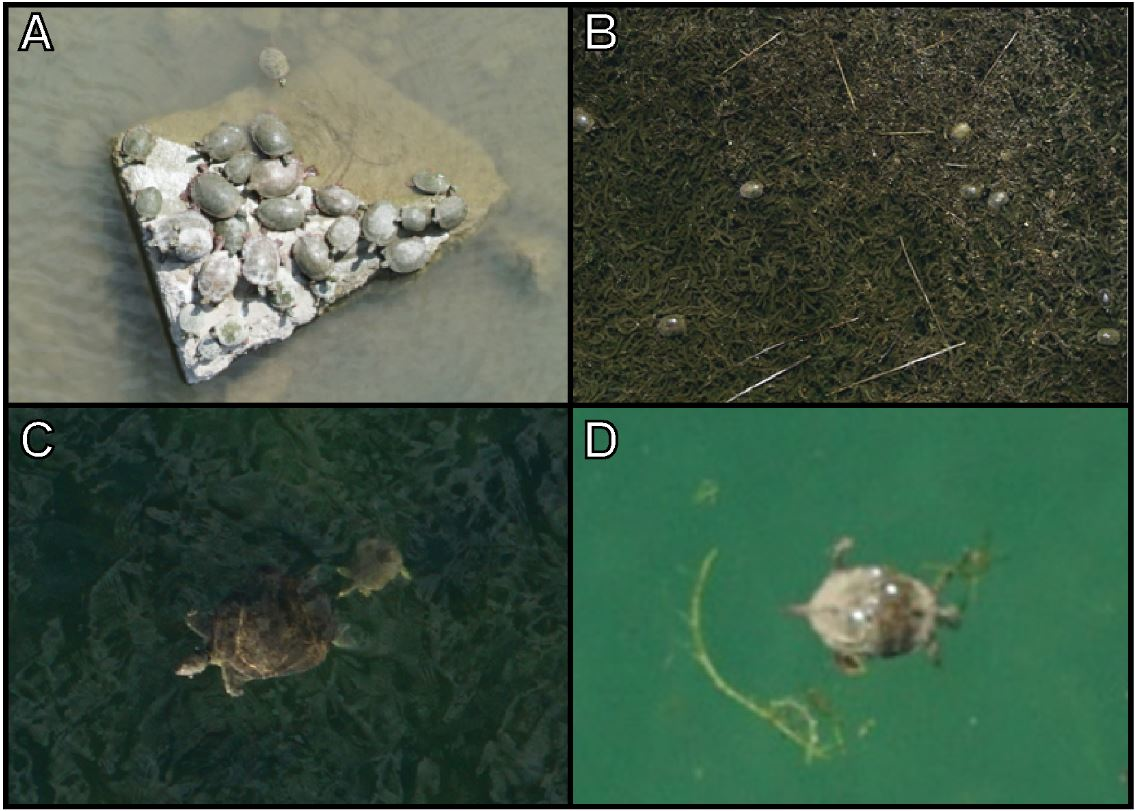

Supplement: S1 Fig — (A) 26 Pseudemys gorzugi basking on one rock at Eagle Pass Golf Course, spillway into Rio Grande, Maverick County. An additional P. gorzugi is seen swimming towards the rock for 27 P. gorzugi total in this image; (B) Subaerial basking of P. gorzugi and Trachemys scripta elegans on aquatic vegetation at Del Rio, San Felipe Springs Golf Course, San Felipe Creek, Val Verde County; (C) Two Apalone spinifera exhibiting courting behaviors in the Rio Grande, spillway below Amistad Dam, Val Verde County; (D) Pseudemys gorzugi seen foraging on aquatic vegetation in TNC Dolan Falls Preserve, Devils River, Dolan Falls, Val Verde County. (TIF) [file pone.0257720.s001.tif]

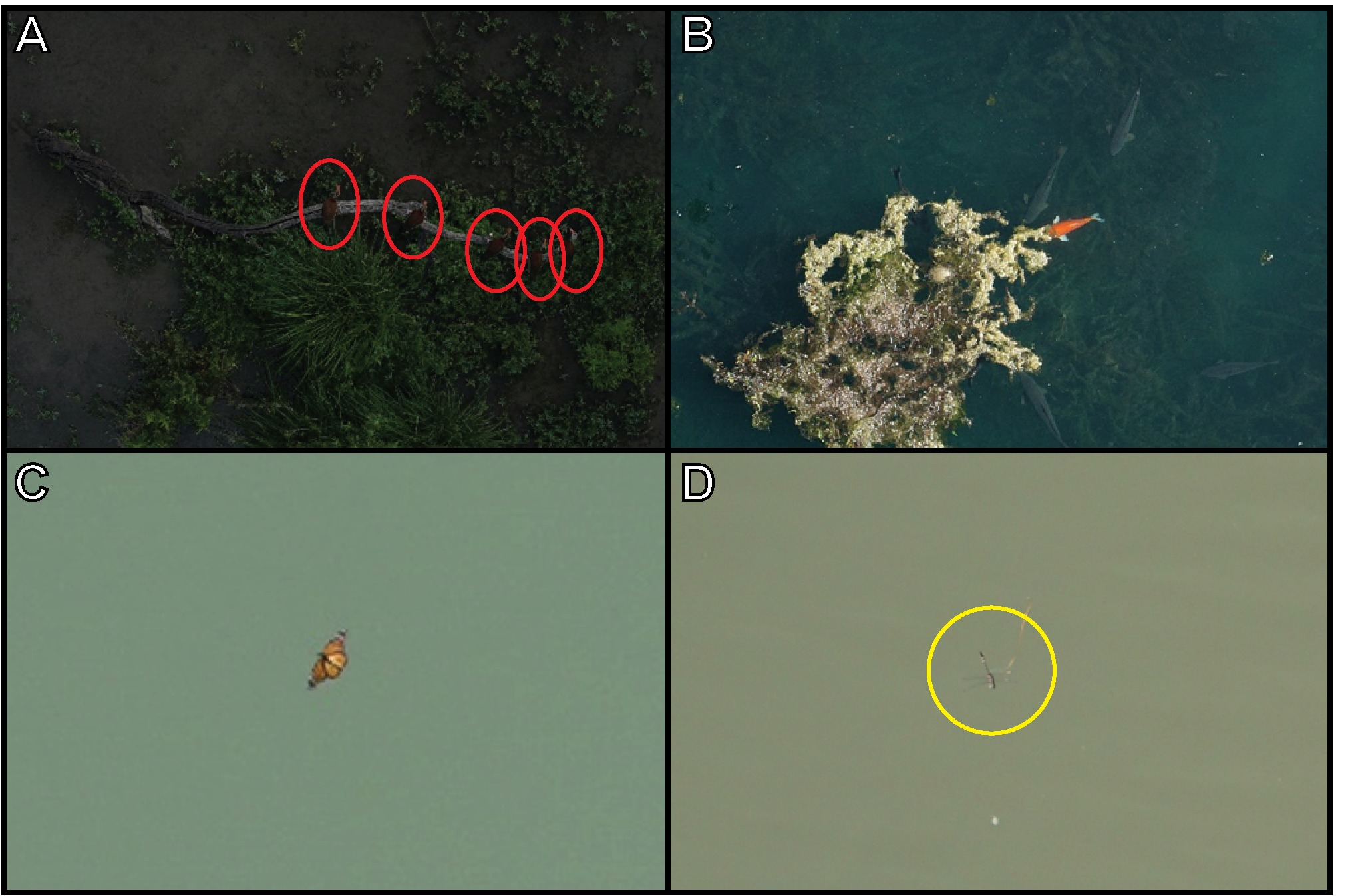

Supplement: S2 Fig — Examples of non-target species that were photographed during surveys, all of which seemed unaffected by the presence of the drone: (A) Five Black-bellied Whistling Ducks (Dendrocygna autumnalis) perched on a log at Fort Clark Springs, Las Moras Creek, Buzzard Roost, Kinney County; (B) Native and introduced fish (Cypriniformes) swimming at Fort Clark Springs, Headwater Pond, Kinney County; (C) Monarch Butterfly (Danaus plexippus) flying over the Pecos River, 0.3 km upstream of confluence with Independence Creek, Crockett County; and (D) Dragonfly (Odonata) flying above the Pecos River, at Pandale Crossing. (TIF) [file pone.0257720.s002.tif]
