# Supplementary material for: A drone-based survey for large, basking freshwater turtle species
Source: PLoS One. 2021 Oct 27;16(10):e0257720. doi: 10.1371/journal.pone.0257720 (PMC8550609; doi:10.1371/journal.pone.0257720)
Supplement: S1 Table — Site information and habitat characterization data for the 42 localities where drone surveys were conducted for Pseudemys gorzugi throughout southwestern Texas, USA. Sites were classified as waterbody type (M = mainstream, T = tributary, R = reservoir), spring-fed (Y or N), presence of aquatic vegetation mats (Y or N), woody debris (Y or N), trees (Y or N) and shoreline vegetation of ca. 2 m or greater (Y or N). For all categories Y = yes and N = no. Site numbers correspond to Table 1 and Fig 1. (DOCX) [file pone.0257720.s003.docx]

| **Site #** | **County** | **Site** | **Latitude** | **Longitude** | **Waterbody Type** | **Spring-fed** | **Aquatic Vegetation** | **Woody Debris** | **Trees** | **Shoreline Vegetation** |
| --- | --- | --- | --- | --- | --- | --- | --- | --- | --- | --- |
| 1 | Pecos | Pecos River, at US Hwy 190 crossing | 30.90516 | -101.88083 | M | N | Y | Y | Y | Y |
| 2 | Pecos | Pecos River, at Texas Rock Rd (Crockett Co Rd 306) | 30.78851 | -101.83502 | M | N | Y | Y | Y | N |
| 3 | Pecos | Pecos River, at I-10 crossing | 30.71808 | -101.80954 | M | N | N | N | Y | Y |
| 4 | Pecos | Pecos River, at TX Hwy 290 crossing | 30.65960 | -101.77022 | M | N | N | N | Y | Y |
| 5 | Terrell | TNC Independence Creek Preserve, Lower Lake | 30.46955 | -101.80131 | T | Y | Y | Y | Y | N |
| 6 | Terrell | TNC Independence Creek Preserve, raceway below Upper Lake | 30.46736 | -101.80181 | T | Y | Y | Y | Y | N |
| 7 | Crockett | Pecos River, 0.8 river km upstream of confluence with Independence Creek | 30.45259 | -101.71940 | M | N | N | N | Y | Y |
| 8 | Terrell | Independence Creek, at County Road crossing | 30.45026 | -101.73124 | T | Y | N | N | Y | N |
| 9 | Crockett | Pecos River, 0.3 river km upstream of confluence with Independence Creek | 30.44767 | -101.72119 | M | N | Y | Y | Y | Y |
| 10 | Val Verde | Pecos River, at Pandale crossing | 30.13120 | -101.57450 | M | N | N | N | Y | Y |
| 11 | Val Verde | TNC Dolan Falls Preserve, Devils River, upstream of confluence with Dolan Creek | 29.89387 | -100.99561 | T | Y | Y | Y | Y | Y |
| 12 | Val Verde | TNC Dolan Falls Preserve, Dolan Creek, near confluence with Devils River | 29.88591 | -100.99292 | T | Y | Y | Y | Y | Y |
| 13 | Val Verde | TNC Dolan Falls Preserve, Devils River, Dolan Falls | 29.88385 | -100.99397 | T | Y | Y | Y | Y | Y |
| 14 | Val Verde | Rio Grande, near Langtry | 29.80564 | -101.55088 | M | N | N | N | Y | Y |
| 15 | Val Verde | Pump Canyon, Langtry | 29.80343 | -101.56750 | T | Y | N | Y | Y | N |
| 16 | Val Verde | Pecos River, near confluence with Rio Grande | 29.70431 | -101.36667 | M | N | N | N | N | N |
| 17 | Val Verde | Lake Amistad, Rough Canyon | 29.57490 | -100.97809 | R | N | N | N | N | N |
| 18 | Val Verde | Lake Amistad, Box Canyon | 29.52420 | -101.17585 | R | N | N | N | N | N |
| 19 | Val Verde | Rio Grande, spillway below Amistad Dam | 29.44737 | -101.05667 | M | N | N | N | Y | Y |
| 20 | Val Verde | Rio Grande, weir below Amistad Dam | 29.42455 | -101.04118 | M | N | N | N | Y | Y |
| 21 | Val Verde | Rio Grande, near Lugo property | 29.37719 | -101.01348 | M | N | N | N | Y | Y |
| 22 | Val Verde | Del Rio, San Felipe Springs Golf Course, San Felipe Creek | 29.37029 | -100.88526 | T | Y | Y | Y | Y | N |
| 23 | Kinney | Fort Clark Springs, Headwater Pond | 29.30944 | -100.42125 | T | Y | Y | Y | Y | N |
| 24 | Kinney | Fort Clark Springs, Las Moras Creek, near guard station | 29.30740 | -100.41745 | T | Y | N | N | Y | Y |
| 25 | Kinney | Fort Clark Springs, Las Moras Creek, upstream of golf pro shop | 29.29043 | -100.42386 | T | Y | N | N | Y | N |
| 26 | Kinney | Fort Clark Springs, Las Moras Creek, Buzzard Roost | 29.28034 | -100.42076 | T | Y | Y | Y | Y | Y |
| 27 | Maverick | Eagle Pass Golf Course, spillway into Rio Grande | 28.70416 | -100.51046 | M | N | Y | Y | Y | N |
| 28 | Maverick | Rio Grande, along Eagle Pass Golf Course | 28.70294 | -100.51089 | M | N | N | N | Y | Y |
| 29 | Maverick | Eagle Pass Golf Course, settling pond along Rio Grande | 28.70146 | -100.50979 | M | N | N | N | N | N |
| 30 | Webb | Lake Casa Blanca International State Park, near El Ranchito pavillion | 27.54447 | -99.44098 | R | N | N | N | Y | Y |
| 31 | Webb | Lake Casa Blanca International State Park, fishing pier | 27.53861 | -99.43475 | R | N | N | N | N | Y |
| 32 | Webb | Rio Grande, Laredo, near water treatment center | 27.52372 | -99.52431 | M | N | N | N | Y | Y |
| 33 | Webb | Rio Grande, Laredo, near international railroad bridge crossing | 27.49835 | -99.51674 | M | N | N | N | Y | Y |
| 34 | Webb | Rio Grande, near El Cenizo | 27.33117 | -99.51195 | M | N | N | N | Y | Y |
| 35 | Zapata | Rio Grande, near San Ygancio | 27.04330 | -99.44496 | M | N | N | N | Y | Y |
| 36 | Starr | Falcon State Park, Falcon Lake | 26.58179 | -99.15259 | R | N | N | N | N | N |
| 37 | Starr | Rio Grande, spillway below Falcon Dam | 26.54608 | -99.17093 | M | N | N | N | Y | Y |
| 38 | Starr | Rio Grande, near Chapeno | 26.53233 | -99.15546 | N | N | Y | Y | Y | Y |
| 39 | Starr | Rio Grande, near Salineño | 26.51429 | -99.11662 | M | N | N | Y | Y | Y |
| 40 | Hidalgo | Rio Grande, near National Butterfly Center | 26.16934 | -98.36742 | M | N | Y | Y | Y | Y |
| 41 | Cameron | Rio Grande, downstream of TNC Southmost Preserve | 25.85462 | -97.37676 | M | N | N | N | Y | Y |
| 42 | Cameron | Rio Grande, near TNC Southmost Preserve Office | 25.85008 | -97.39865 | M | N | N | N | Y | Y |
