# Supplementary material for: A drone-based survey for large, basking freshwater turtle species
Source: PLoS One. 2021 Oct 27;16(10):e0257720. doi: 10.1371/journal.pone.0257720 (PMC8550609; doi:10.1371/journal.pone.0257720)
Supplement: S1 Protocol — Protocol detailing drone flight preparations, instructions for flight, field checking data, and image analysis that was used for this study. This can be used as a guideline and modified to be appropriate for different equipment, environments, and sampling conditions. (DOCX) [file pone.0257720.s004.docx]

**Day Before Flight**

- Charge sampling equipment (drone batteries, digital camera, remote controller, tablet, and laptop)
- Program flights with a maximum speed of 2.2 m/s, altitude of 30 m, and frontal overlap of 50–60% over 1.25 ha area; flight lines should be perpendicular to flow in lotic systems and encompass the entire survey area (water body and 10 m shore boundary)
- Clear SD and microSD cards
- Open the Config file in the microSD card set the file name convention to SONY, set trigger mode to time, specify the time interval at 1 s, and save changes
- Check the weather to ensure conditions will be adequate for flight (wind less than 8 m/s no rain)

**Pre-flight**

- Extend drone propeller arms and firmly lock in place; insert drone batteries
- Raise up GPS arms, ensure GPS arrows are correctly oriented, and that all cable connections are secure
- Take off drone propeller covers and unfold propellers
- Ensure camera is on Manual and F-stop is 6.3, ISO is 320, shutter speed is 1/1000, and that a clear SD card is inserted; focus digital camera to 29 m
- Attach digital camera to the drone with the gimbal
- Detach power cord from the GeoSnap Express and insert microSD card; insert camera trigger cable into the digital camera
- Turn on tablet and open Maps Made Easy app
- Turn on remote controller
- Power on drone
- Position the gimbal so that the camera is level to the ground and lock it in place
- Open saved mission and adjust mission if needed; double check flight line and parameters
- Make sure that the app, remote controller, and drone are all connected
- Upload mission
- Reattach the power cord for the GeoSnap Express; ensure the GeoSnap control unit is powered on (blue light is on), the GPS is fixed (green light is rapidly flashing), and that the camera is taking pictures (shutter release can be heard)
- Initiate flight

**Upon Landing**

- Once propellers have come to a complete stop, press and hold white onboard button on GeoSnap for 3 s to cease photo triggering
- Power off digital camera
- Power off drone
- Power off remote controller
- Press okay, that mission is complete on Maps Made Easy app and exit Maps Made Easy app
- Power off tablet
- Fold drone propellers, put on covers, and collapse arms
- Fold down three GPS antennas

**Field Checking Data**

*Digital Camera*

- Remove SD card from camera and open the file in File Explorer
- Ensure that photos were taken during the duration of the flight time, the images are in focus, and the camera settings were as desired; note the number of images acquired
- Close out of file and safely eject SD card

*GeoSnap Express*

- Remove microSD card from control unit
- Insert into microSD adapter and open the IMG file in the computer
- Ensure image labels and total number match those of the digital camera; ensure that latitude and longitude data is populated for all photos; initial photos will likely not have a latitude and longitude as camera triggering begins before the GPS is fixed, however, as these photos were taken before the drone was launched this will not affect analysis
- Close out of file and safely eject microSD card

**Image Analysis**

- Manually sort through digital photos noting the species of each turtle present and the corresponding photograph number; copy each photograph containing a turtle to a new folder
- Download IMG file from the microSD to computer and open in Excel; make sure “all files” is selected when navigating to the file
- Select “my data has headers” box, start import at row 4, and chose space delimited; click finish
- Delete all columns except for image, latitude, and longitude
- Delete all rows except for the photograph numbers that correspond to those containing turtles in the new folder
- Save the excel file in csv format and close the file
- Open Google Maps and select “Your places” in the menu options
- Click “Maps” and click “Create Map”
- Assign a map title in the Untitled Map field
- Click Import and navigate to the .csv file
- Check the “lat” box and assign this as latitude; check the “long” box and assign this as longitude; click continue
- Select the “image” box and click finish
- Customize base map and icons as desired
- Use the image number labels to identify which photographs are in close proximity; examine the digital photographs, noting the species, size, sex, unique markings, activity, and location of the turtles to determine if there are any duplicates; once duplicate turtles have been identified, determine the counts for each species
